# Factors affecting pretransplant muscle strength in allogeneic stem cell transplant candidates prior transplantation

**DOI:** 10.1007/s00520-024-09140-8

**Published:** 2025-01-10

**Authors:** Matthias Limbach, Rea Kuehl, Maximilian Koeppel, Peter Dreger, Thomas Luft, Martin Bohus, Joachim Wiskemann

**Affiliations:** 1 Department of Medical Oncology, Heidelberg University Hospital and National Center for Tumor Diseases Heidelberg, a partnership between German Cancer Research Center (DKFZ) and University Medical Center Heidelberg, Im Neuenheimer Feld 460, 69120 Heidelberg, Germany; 2 Institute of Sports and Sport Science, Heidelberg University, Im Neuenheimer Feld 700, Heidelberg, Germany; 3 Department of Medicine V, Heidelberg University Hospital, Heidelberg, Germany; 4 Central Institute of Mental Health, Mannheim, Germany

**Keywords:** Exercise, Allogeneic stem cell transplantation, Oncology, Muscle strength, Pretransplantation

## Abstract

**Purpose:**

Physical performance is crucial for prognosis after allogeneic hematopoietic stem cell transplantation (allo-HCT). Cardiorespiratory fitness has already been shown to have prognostic value, and there is increasing evidence that muscle strength and associated parameters (e.g., sarcopenia) are also of clinical relevance. Therefore, there is a need for the quantification of muscle strength and defining risk factors for reduced performance values.

**Methods:**

Maximal voluntary isokinetic (MVIC) and isometric (MIPT) muscle strength was assessed 2.4 ± 7.1 days prior admission for allo-HCT with a stationary isokinetic testing machine (IsoMed2000). We calculated percentiles for knee extension and hip flexion using healthy reference values. Regression models were used to identify predictors for reduced muscle strength including gender, age, body mass index (BMI), number of previous cardiotoxic therapies, number of previous transplantations, comorbidity index (HCT-CI), hemoglobin level, and physical activity.

**Results:**

Data of 212 patients (male *n* = 143, female *n* = 69), with a mean age of 54.49 ± 11.4, revealed considerably deviations from healthy reference values. Patients were located in the following percentiles: MVIC_Knee_ 37.5 ± 30.3, MVIC_Hip_ 39.5 ± 31.3 and MIPT_Knee_ 22.9 ± 26.5; MIPT_Hip_ 22.6 ± 27.4. Sub-group analyses showed that patients with younger age and male gender possess the highest deviations. Muscle strength values were significantly (*p* < 0.05) influenced by age, female gender, lower BMI, and higher HCT-CI.

**Conclusion:**

Muscle strength is considerably reduced immediately prior to allo-HCT. Identified patient characteristics for reduced muscle strength point to the population that should be primarily targeted with exercise respectively resistance training interventions prior to allo-HCT to contribute to a well prepared transplant candidate.

**Trial registration:**

NCT01374399.

## Introduction

Allogeneic stem cell transplantation (allo-HCT) is a curative therapy for a variety of malign hematopoietic disorders. Although remarkable therapeutic successes due to enhanced treatment techniques and supportive care lead to an improved outcome and even older or comorbid patients can be treated, there is a substantial risk of therapy-related morbidity and mortality [1, 2]. Because of the intensive and demanding treatment, the determination of appropriate candidates for allo-HCT is crucial, as is the identification of risk factors that potentially influence the outcome [3]. It is assumed that physical performance in general is a relevant predictive factor [3]. Research in this area has primarily focused on pretransplant cardiorespiratory fitness (CRF), showing that CRF is a potentially predictive indicator for symptom burden and mortality in allo-HCT-candidates [4]. Besides CRF, it has been described that strength performance also appears to be crucial and of prognostic relevance in cancer patients [5, 6]. In allo-HCT candidates, it has been revealed that significant muscle weakness and limitations in muscle strength exist during the course of allo-HCT [7–9]. Furthermore, sarcopenia, defined as a loss of muscle mass and muscle strength, has become apparent to be associated with a significantly increased risk of poor outcome in allo-HCT candidates [10–12]. Thus, it is reasonable that muscle strength could be a crucial influencing factor that needs to be investigated more comprehensively in the light of allo-HCT. However, in the existing literature, muscle strength performance has been primarily assessed by hand-held dynamometry and functional testing procedures, whereas gold standard procedures like stationary isokinetic dynamometry have not been applied so far. Within our RCT, aiming to investigate the effects and biological mechanisms of an 1-year exercise intervention on side effects complications and survival in allo-HCT patients during and after transplantation [13], we assessed muscle strength prior transplantation by using the gold-standard approach of stationary isokinetic dynamometry. Therefore, the aim of this manuscript is to report isokinetic and isometric muscle strength in patients prior allo-HCT and to identify potential influencing factors in a cross-sectional analysis.

## Patients and methods

### Setting and patients

We analyzed baseline data from 212 allo-HCT patients from the study *Physical Exercise Training versus Relaxation in Allogeneic stem cell transplantation* (PETRA) [13]. All patients, scheduled for an allo-HCT at the Heidelberg University Clinic, of age ≥ 18 years and able to understand and follow the study protocol, were eligible for enrollment in the PETRA trial. Exclusion criteria were inability to walk or stand, instable bone lesions, severe neurological deficiencies, severe cardiac or cardiovascular diseases, and severe overall pulmonary insufficiency. Cross-sectional data were obtained prior to admission for allo-HCT. The study has been approved by the ethic committees of the Ethic Committee II of the University of Mannheim (number 2009–349 N-MA) and the Ethic Committee of the University of Heidelberg (number S-021/2011) and is registered at ClinicalTrials.gov (NCT01374399). All patients provide written informed consent.

### Determination of the muscle strength

Isokinetic and isometric muscle strength was measured by using the IsoMed 2000-system B-series version (D&R Ferstl GmbH, Hemau, Germany). The reliability and validity of this measurement has been reported in serval studies [14–16], and isometric and isokinetic assessments are considered as gold standard method to measure muscle strength [5]. Furthermore, it was successfully applied in various cancer populations [17–20]. Our test protocol includes the assessment of the maximal voluntary isometric contraction (MVIC) and the maximal isokinetic peak torque (MIPT) of knee extensors and hip flexors. The measured torques are reported as Newton meter (Nm).

During test protocols, both the subject and the instructor were able to see the strength curve on the monitor. Subjects were given verbal encouragement to generate the highest possible strength. To minimize extraneous body movements, subjects were fixed by straps (thigh, pelvic, torso) and used handlebars during assessment of extensors and flexors of the knee. Each torque artifact resulting from deceleration, which often exceeds the true peak torque, was removed by using a filter; only gravity-corrected data were used for analysis.

### Assessment of MVIC

Maximal voluntary isometric contraction (MVIC) was tested bilaterally for knee extensor (angle position 36°) and hip flexor (angle position 33°), which consistently were the strongest angle positions each. Patients were requested to exert maximum force against an insurmountable resistance and to hold it for 6 s.

### Assessment of MIPT

Maximal isokinetic peak torque (MIPT) was assessed bilaterally for extensors and flexors of the knee and hip. Range of motion for isokinetic measurements varied from 10° to 90° flexion in the knee (straight leg is 0°) and from 10° to 100° in the hip (straight leg in dorsal position is 0°). Patients were instructed to move the machine arm for 10 repetitions as firmly and as fast as possible in the alternating direction between extension and flexion with constant angular velocity (60°/s). The highest torque value of all cycles at considered joint side was analyzed.

### Descriptive and influencing variables for muscle strength

We investigated potentially influencing factors for MVIC and MIPT. Congruent with our previous analysis on cardiorespiratory fitness [21] these were age, gender, body mass index (BMI), total number of transplantations (allogeneic and autologous, n_Transplantations), number of previous cardiotoxic therapies (n_cardiotox), months between last treatment to allo-HCT (chemotherapies respectively cytostatics, radiotherapies or immunotherapies that were given for the treatment of the specific disease before admission to the allo-HCT) (T_Therapies), the hematopoietic cell transplantation-specific comorbidity index (HCT-CI), physical activity, and hemoglobin levels. Further, we gathered information on primary hematological malignancies and the proportion of myeloid and lymphatic malignancies, Karnofsky Performance Status (KPS), and months between primary diagnoses to allo-HCT (T_Diagnosis).

Potentially influencing variables and clinical data were extracted from patient’s medical records. BMI was calculated as body weight divided by square of height in meters. HCT-CI was determined according to guidelines and separated into low to intermediate risk group (< 3) and high risk group (≥ 3) [22]. We used a modified version of the SQASH [23] questionnaire to assess patients self-reported frequency (days per week), duration (hours per day), and intensity (light, moderate, partly vigorous, mainly vigorous) and type of physical activity (walking, cycling, and other exercise/sports activities) in a typical week in the year before diagnosis. The degree of physical activity was operationalized with metabolic equivalent of task (MET). MET hours per week (MET × h/week) were calculated by summing the average of self-reported hours per week spent in walking, cycling, or other physical activities. Hemoglobin (g/dl) level was measured venously on the day of muscle strength assessment. Lastly, we calculated the area under the curve values for hemoglobin levels over the last 3 months prior allo-HCT (Hemoglobin_auc).

### Statistical analyses

Clinical and demographic data were reported by descriptive analysis and are reported as mean ± SD and range. Expected muscle strength values were calculated by the formula of Danneskiold-Sam (2009) [24] for healthy individuals considering age and gender distribution. Muscle strength values were presented as percentiles to illustrate the deviation to the healthy reference collective, whereas the 50th percentile represents the median of the reference sample.

Hemoglobin level over the last 3 months was calculated as area under the curve (AUC) score using the trapezoidal rule [25]. To identify determinants of muscle strength prior allo-HCT, a multivariate regression analysis was conducted. In consideration of multicollinearity, we aimed to identify the most relevant determinants. The following independent variables were entered into the regression model simultaneously: age, gender, BMI, n_Transplantations, n_Cardiotox, t_Therapies, HCT-CI, physical activity, and Hemoglobin_auc. We chose the muscle strength of the quadriceps muscle as a representative value for loads of activities of daily living. The values of MVIC and MIPT were defined as dependent variable: MVIC_Knee_ and MIPT_Knee_. Significant level was set at *p* < 0.05. Data were analyzed using IBM SPSS v. 26 and R version 4.0.

## Results

**Fig. 1:**
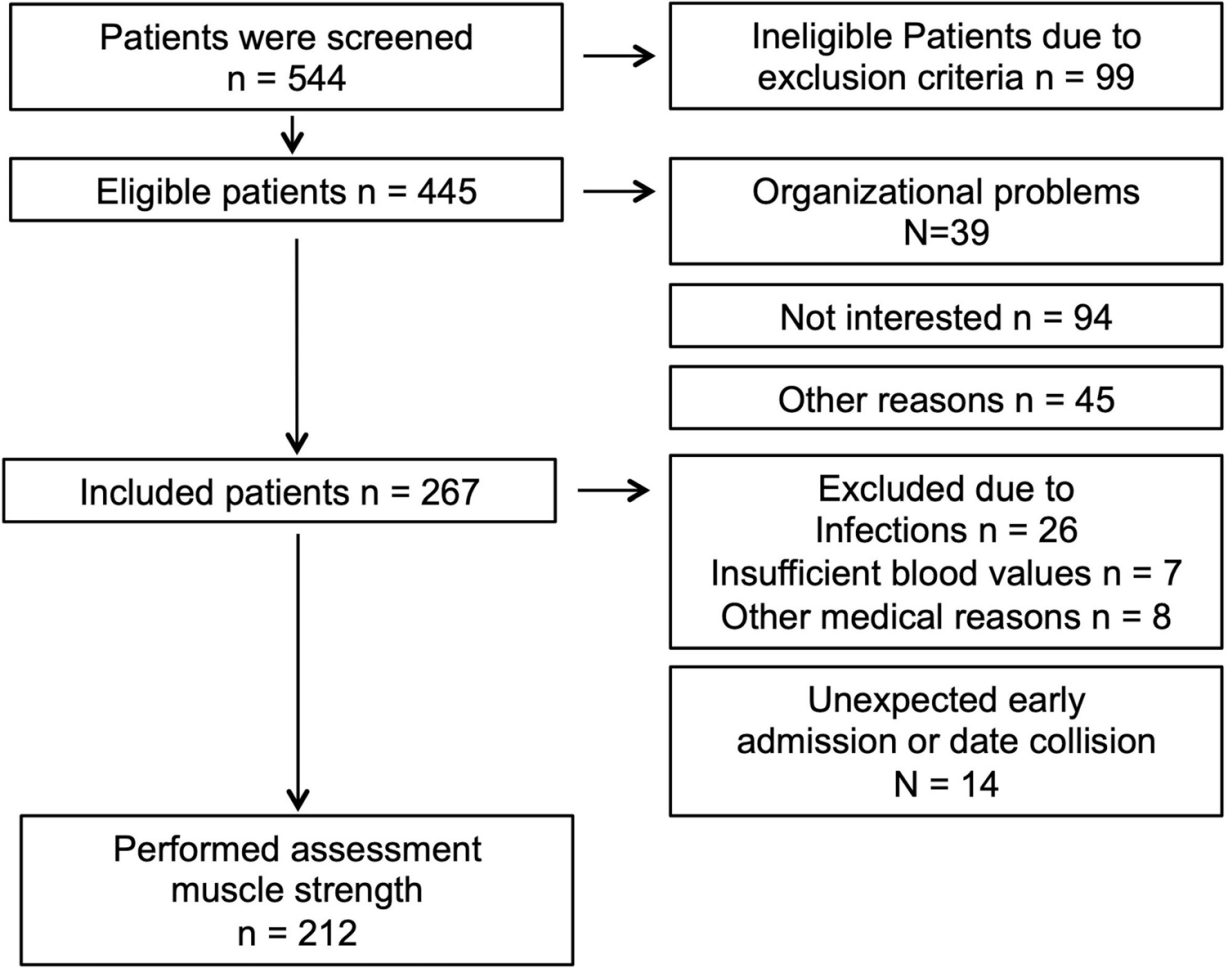
Patient flow chart showing the number of patients who were available for muscle strength assessment

Five hundred forty-four patients were screened, and *n* = 99 were ineligible for the study due to exclusion criteria (18.2%). From *n* = 445 (81.8%), eligible patients (*n* = 178, 40%) did not participate due to no interest (*n* = 94, 52.8%), organizational problems (postponement, no exercise testing capacity available due to short notice admission to allo-HCT; *n* = 39, 21.9%) and *n* = 45 (25.3%) because of other reasons. Between 9/2011 and 4/2017, 267 patients were included in the study of whom *n* = 212 participated in the assessments of MVIC and MIPT. Major reasons for not being able to perform this strength testing can be seen in Fig. 1. No (serious) adverse events occurred.

Clinical data and demographics of study participants who performed MVIC and MIPT are described in Table 1. Patients had a mean age of 54.49 ± 11.4 years, and the main diagnosis was acute leukemia (AML, 29.1%). Participants were on average 36.2 ± 48 months after main diagnosis, and 54 patients (25.6%) received one or more previous transplantations. Mean hemoglobin at the day of physical assessment was 11.6 ± 1.9 (g/dl), and hemoglobin level during the last 3 months (Hemoglobin_auc) was 11.3 ± 1.9 (g/dl) and therefore below the normal range. On average, the patients performed the measurements 2.4 ± 7.1 days prior admission to allo-HCT.

**Table 1:** Baseline descriptive data

	*n*	Mean	SD	Range
Age (yr) at diagnosis	212	54.49	11.4	18–75
Gender, *n* (%)	212 (100)			
Males	143 (67.5)			
Females	69 (32.5)			
BMI (kg/m^2^)	212	26.41	4.5	17–45
Diagnosis, *n* (%)	210 (100)			
AML	61 (29.1)			
ALL	12 (5.7)			
CLL	39 (18.6)			
MM	24 (11.4)			
CML/MPS	18 (8.6)			
MDS	19 (9.1)			
Other lymphomas	34 (16.2)			
Other	3 (1.4)			
Missing	2 (0.9)			
Time from diagnosis to alloHSCT (month) Missing	210 2	36.2	48.0	1–328
Time between exercise test to admission alloHSCT (days)	212	2.4	7.1	0–47
HCT-CI (*n*)	200	0.91	1.4	0–7
< 3	173			
≥ 3	27			
Missing	12			
KPS (*n*)	207	91.48		100–70
< 90	23			
≥ 90	184			
Missing	5			
Hemoglobin value day of testing (g/dl)	212	11.6	1.9	6.8–17.0
Remission status prior alloHSCT (%)	211 (100)			
CR	75 (35.5)			
PR	77 (36.5)			
No CR	55 (26.1)			
Unknown	4 (1.9)			
Missing	1 (0.5)			
Smokers (%)	196 (100)			
Current	9 (4.2)			
Ever	68 (32.1)			
Year before diagnosis	29 (13.7)			
Never	98 (46.2)			
Missing	8 (3.8)			
Time since last therapy (month)	212	2.9	7.5	0–88
Physical activity (MET h/week) Missing	208 4	7.9	15.5	0–99.5
Hemoglobin last 3 months (g/dl) Missing	209 3	11.3	1.9	6.5–15.7
Amount of cardiotoxic agents	210	1.5	1.4	0–9
Number of previous transplantations Received one or more transplantation Missing	211 54 1	0.32	0.6	0–3

*N* Newton, *n* number of patients, *BMI* body mass index, *HCT-CI* comorbidity index by Sorror, *KPS* Karnofsky Performance Status, *allo-HCT* allogeneic stem cell transplantation, *CR* complete remission, *PR* partial remission, *AML* acute myeloid leukemia, *ALL* acute lymphocytic leukemia, *MM* multiple myeloma, *CML* chronic myeloid leukemia, *MPS* myeloproliferative syndrome, *MDS* myelodysplastic syndrome

### Description of muscle strength values for MVIC

Muscle strength assessments demonstrated mean values for MVIC_Knee_ (*n* = 212) of 159.7 ± 48.3 Nm. Men (*n* = 143) reached 178.4 ± 45.6 Nm, whereas 120.9 ± 25.6 Nm were measured for the women (*n* = 69). Mean values for MVIC_Hip_ (*n* = 203) were 129.72 ± 44.48 Nm. Men (*n* = 140) had values of 143.82 ± 43.2 Nm, and women (*n* = 63) achieved values of 92.36 ± 28.56 Nm.

### Description of muscle strength values for MIPT

For MIPT_Knee_, we obtained mean values of 118.6 ± 40.3 Nm (*n* = 211) and a coefficient of variance (CV) of 34%, with males (*n* = 143) reaching 134.0 ± 40.3 Nm and females (*n* = 68) achieving 86.7 ± 20.7 Nm. MIPT_Hip_ (*n* = 201) measured 100.1 ± 38.81 Nm with a CV of 38.9%. Men (*n* = 139) obtained values of 110.54 ± 37.1 Nm and women (*n* = 62) of 76.41 ± 22.7 Nm.

### Muscle strength (MVIC and MIPT) in relation to healthy reference values

On average, assessed patients were located between the 37th and 40th reference percentile (MVIC_Knee_ 37.5 ± 30.3, MVIC_Hip_ 39.5 ± 31.3). The proportion of patients below the median (50th percentile) of the reference group is for both MVIC_Knee_ and MVIC_Hip_ at 66%. On average, the reference percentiles results for MIPT_Knee_ were 22.9 ± 26.5, respectively, 22.6 ± 27.4 for MIPT_Hip_, resulting in a proportion of 84% (MIPT_Knee_) and 83% (MIPT_Hip_) under the 50th reference percentile.

**Fig. 2:**
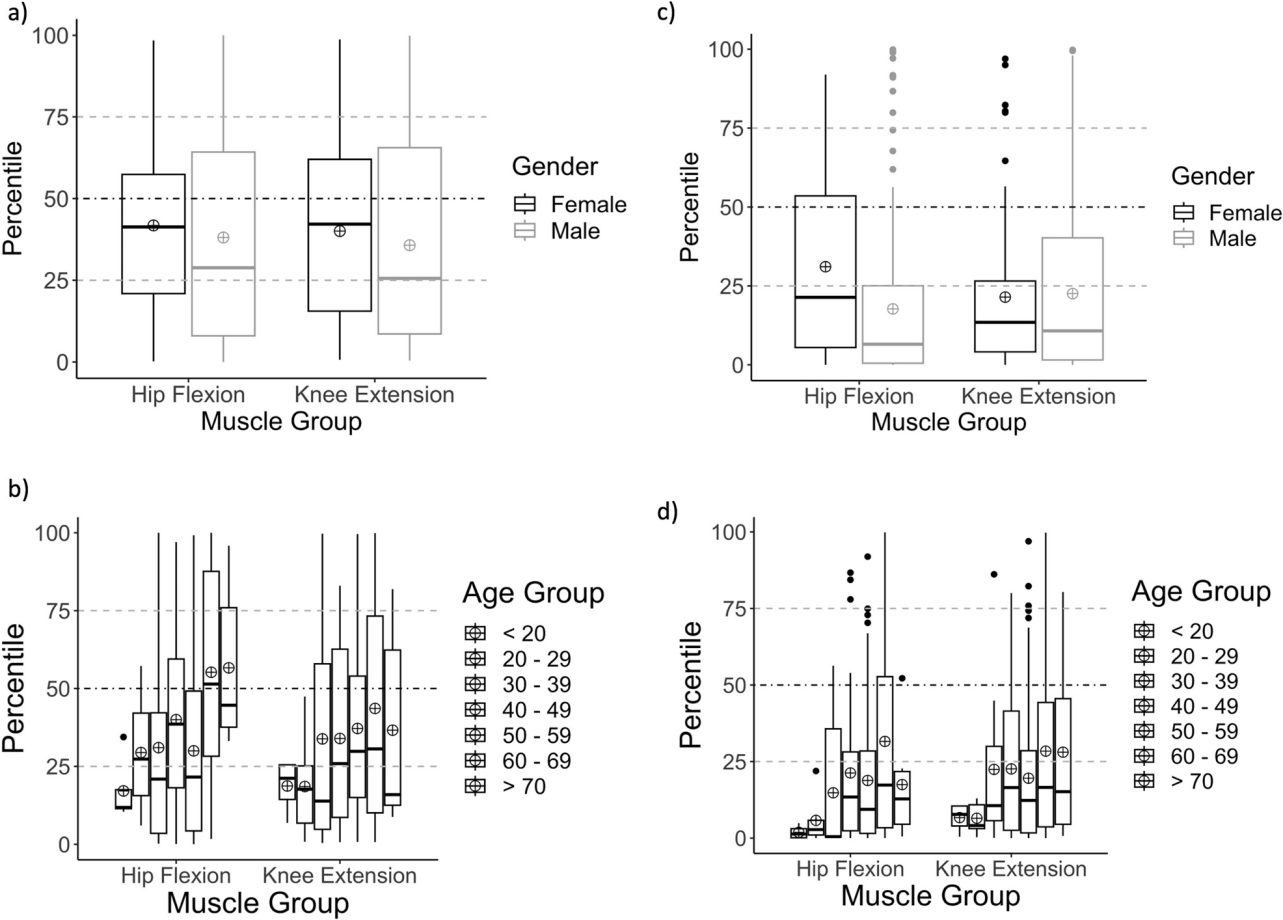
Reference percentiles of MVIC values for gender (**a**) and age group (**b**) and reference percentiles of MIPT 60°/s values for gender (**c**) and age group (**d**). For (**b**) and (**d**) groups are displayed by age from left (< 20 years) to right (> 70 years). **–**, median line; ⨁, mean

Figure 2 demonstrates the deviation of the measured MVIC values as percentiles from the calculated healthy reference values depending on (a) male and female gender and for the different (b) age groups. In general, men showed a higher deviation than women in all investigated aspects. Concerning age, the younger patients, in particular < 20 years and 20–29, reveal the higher deviations and seem to be most affected in their physical strength performance.

Figure 2c and d shows the deviation of the measured MIPT values from the calculated healthy reference values according to the (Fig. 2c) female and male gender and to the different (Fig. 2d) age groups. With regard to the muscle groups, the values of “hip” and “knee” demonstrate higher muscle performance of women. Men demonstrate a higher deviation from healthy reference values than women in the hip flexion, whereas the mean values in the knee extension are nearly congruent for both genders. Taking patients’ age into account, all age groups show a strong limitation of performance. Patients with ages < 20 reveal the highest deviation in hip values, but also the ages of 20 and 39 show substantial deviations. Focusing on the knee values, the younger patients between < 20 and the ages of 20 and 29 display the greatest difference compared to their healthy peers.

### Factors affecting muscle strength (MVIC_Knee_ and MIPT_Knee_)

Regression analysis for MVIC_Knee_ with 192 patients providing a full dataset of the integrated variables shows that higher age, female gender, and lower BMI are significant determinants for low MVIC. The explaining variance is 42.3%. The analysis for MIPT_Knee_ reveals that higher age, female gender, lower BMI, and higher HCT-CI are significant factors with an explaining variance of 46.7% (Table 2).

**Table 2:** Multiple regression of the determinants of muscle strength: MVIC_Knee_ and MIPT_Knee_ for 60°/s

MVIC_Knee_ (Nm) *R*^2^ = 42.3%, *n* = 192, *p* = 0.000			
	*β*	*p*	95% CI
Gender	− 0.570	0.000	− 71.220; − 47.308
Age	− 0.249	0.000	− 1.564; − 0.562
BMI	0.173	0.004	0.605; 3.137
T_Therapies	0.111	0.072	− 0.104; 2.382
n_cardiotox	− 0.109	0.115	− 8.709; 0.954
HCT-CI	− 0.091	0.118	− 7.163; 0.814
Physical activity	0.039	0.528	− 0.255; − 0.495
n_Transplantations	0.032	0.638	− 8.222; 13.389
Hemoglobin_auc	0.036	0.546	− 2.152; 4.058
MIPT_Knee_ (Nm) *R*^2^ = 46.7%, *n* = 191, *p* = 0.000			
	*β*	*p*	95% CI
Gender	− 0.570	0.000	− 59.023; − 39.810
Age	− 0.278	0.000	− 1.388; − 0.0583
BMI	0.174	0.003	− 0.550; 2.574
T_Therapies	0.080	0.177	− 0.311; − 1.677
n_cardiotox	− 0.093	0.163	− 6.615; 1.121
HCT-CI	− 0.114	0.043	− 6.496; − 0.111
Physical activity	0.070	0.232	− 118; 482
n_Transplantations	0.032	0.625	− 6.493; 10.784
Hemoglobin_auc	0.113	0.052	− 0.018; 4.961

*MVIC*, maximal voluntary isometric contraction; *MIPT*, maximal isokinetic peak torque; *BMI*, body mass index (kg/m^2^); *T_Therapie*, months between last treatment to allo-HCT; *n_cardiotox*, number of previous cardiotoxic therapies; *HCT-CI*, comorbidity index by Sorror; *n_Transplantations*, total number of transplantations; *CI*, confidence interval; physical activity (MET); *Hemoglobin_auc*, hemoglobin levels over the last 3 months (g/dl) prior allo-HCT; *Nm*, Newton meter

### Factors affecting muscle strength deviation in comparison to reference values (MVIC_Knee_ and MIPT_Knee_)

Regression analysis for MVIC_Knee_ with 177 patients shows that lower BMI and the HCT-CI were significant determinants for low MVIC. The explained variance (*R*2) was 13.1%. Analysis for MIPT_Knee_ reveals also that lower BMI values and higher HCT-CI values were significantly explaining factors with an overall *R*2 19.3% (Table 3).

**Table 3:** Multiple regression of the determinants of muscle strength deviation from reference values: MVIC_Knee_ and MIPT_Knee_ for 60°/s

MVIC_Knee_ (Nm) *R*^2^ = 13.1%, *n* = 177, *p* = 0.005			
	*β*	*p*	95% CI
Gender	− 0.043	0.583	− 71.220; − 47.308
Age	0.129	0.096	− 1.564; − 0.562
BMI	0.165	0.033	0.605; 3.137
T_Therapies	0.151	0.071	− 0.104; 2.382
n_cardiotox	− 0.117	0.204	− 8.709; 0.954
HCT-CI	− 0.160	0.033	− 7.163; 0.814
Physical activity	− 0.034	0.675	− 0.255; − 0.495
n_Transplantations	0.129	0.155	− 8.222; 13.389
Hemoglobin_auc	0.056	0.477	− 2.152; 4.058
MIPT_Knee_ (Nm) *R*^2^ = 19.3%, *n* = 184, *p* = 0.000			
	*β*	*p*	95% CI
Gender	0.134	0.059	− 59.023; − 39.810
Age	0.124	0.053	− 1.388; − 0.0583
BMI	0.224	0.002	0.550; 2.574
T_Therapies	0.076	0.305	− 0.311; − 1.677
n_cardiotox	− 0.128	0.136	− 6.615; 1.121
HCT-CI	− 0.167	0.019	− 6.496; − 0.111
Physical activity	0.107	0.151	− 118; 482
n_Transplantations	0.003	0.937	− 6.493; 10.784
Hemoglobin_auc	0.135	0.066	− 0.018; 4.961

*MVIC*, maximal voluntary isometric contraction; *MIPT*, maximal isokinetic peak torque; *BMI*, body mass index (kg/m^2^); *T_Therapie*, months between last treatment to allo-HCT; *n_cardiotox*, number of previous cardiotoxic therapies; *HCT-CI*, comorbidity index by Sorror; *n_Transplantations*, total number of transplantations; *CI*, confidence interval; physical activity (MET); *Hemoglobin_auc*, hemoglobin levels over the last 3 months (g/dl) prior allo-HCT; *Nm*, Newton meter

### Muscle strength in various subgroups

Table 4 shows descriptive muscle strength values for MVIC_Knee/kg_ and MIPT_Knee/kg_ MVIC_Knee_ and MIPT_Knee_ for 60°/s. for various clinical parameters. Overall, no distinctive subgroups could be identified, but slight tendencies can be depicted.

**Table 4:** Muscle strength performance values for clinical subgroups

Variable (*n* MVIC, *n* MIPT)	MVIC_Knee/kg_ (Nm) Mean ± SD	MIPT_Knee/kg_ (Nm) Mean ± SD	MVIC_Knee_ (Nm) Mean ± SD	MIPT_Knee_ (Nm) Mean ± SD
Diagnosis				
AML (*n* = 61)	1.99 ± 0.49	1.49 ± 0.41	156.9 ± 47.2	118.1 ± 40.2
ALL (*n* = 12)	1.91 ± 0.54	1.28 ± 0.28	136.5 ± 45.2	95.6 ± 28.4
CLL (*n* = 39)	2.01 ± 0.45	1.52 ± 0.41	161.9 ± 42.1	123.4 ± 39.2
MM (*n* = 24)	1.84 ± 0.64	1.35 ± 0.45	159.4 ± 60.8	117.3 ± 42.4
CML/MPS (*n* = 18, *n* = 17)	2.22 ± 0.39	1.58 ± 0.27	172.7 ± 37.5	126.3 ± 32.5
MDS (*n* = 19)	1.88 ± 0.53	1.31 ± 0.38	156.8 ± 52.8	108.4 ± 36.3
Other lymphomas (*n* = 34)	2.08 ± 0.51	1.57 ± 0.57	164.2 ± 52.5	124.9 ± 47.3
Other (*n* = 3)	1.66 ± 0.25	1.07 ± 0.29	137.5 ± 30.1	87.5 ± 21.1
Malignancy				
Myeloid (*n* = 101)	2.01 ± 0.49	1.47 ± 0.38	158.8 ± 46.8	116.7 ± 38.0
Lymphatic (*n* = 109)	1.98 ± 0.52	1.48 ± 0.44	160.5 ± 49.9	120.5 ± 42.2
Missing (*n* = 2)				
Received no prior transplantation (*n* = 157, *n* = 156)	1.98 ± 0.48	1.46 ± 0.41	158.7 ± 45.4	117.6 ± 38.6
Received one or more prior transplantations (*n* = 54)	2.02 ± 0.58	1.51 ± 0.44	161.3 ± 56.3	120.7 ± 44.9
KPS				
100% (*n* = 73)	2.07 ± 0.57	1.56 ± 0.47	160.3 ± 52.6	120.2 ± 44.1
90% (*n* = 111)	1.97 ± 0.46	1.46 ± 0.37	159.2 ± 44.7	118.8 ± 37.1
80% (*n* = 22)	1.80 ± 0.45	1.23 ± 0.32	162.2 ± 52.9	110.3 ± 8.3
≤ 70% (*n* = 1)	2.12 ± 0	1.41 ± 0	141.1 ± 0	94.2 ± 0
Missing (*n* = 5)				
HCT-CI				
< 3 (*n* = 173, *n* = 172)	1.99 ± 0.51	1.49 ± 0.42	159.9 ± 49.6	120.3 ± 41.2
≥ 3 (*n* = 27, *n* = 27)	1.98 ± 0.42	1.42 ± 0.37	158.5 ± 44.9	112.5 ± 35.5
Missing (*n* = 12, *n* = 13)				
T_Therapy				
< 3 months (*n* = 163, *n* = 162)	2.00 ± 0.48	1.46 ± 0.39	160.5 ± 45.8	118.3 ± 38.4
3–6 months (*n* = 38)	1.93 ± 0.55	1.49 ± 0.46	157.6 ± 58.1	121.6 ± 47.3
> 6 months (*n* = 14)	2.00 ± 0.63	1.45 ± 0.54	164.5 ± 58.7	122.6 ± 48.2
Missing (*n* = 1, *n* = 2)				
T_Diagnosis				
≤ 6 months (*n* = 73, *n* = 72)	1.96 ± 0.53	1.42 ± 0.42	154.4 ± 46.7	113.6 ± 40.4
≥ 7 months (*n* = 137)	2.01 ± 0.49	1.50 ± 0.41	161.9 ± 49.2	120.8 ± 40.2
Missing (*n* = 2, *n* = 3)				
Physical activity (MET h/week)				
< 7.0 (*n* = 143)	1.96 ± 0.48	1.44 ± 0.40	156.9 ± 47.7	116.1 ± 39.8
≥ 7.0 (*n* = 65)	2.01 ± 0.54	1.56 ± 0.44	166.3 ± 49.1	125.6 ± 40.6
Missing (*n* = 4)				
Hemoglobin last 3 months				
≤ 10 (g/dl) (*n* = 67, *n* = 66)	1.91 ± 0.50	1.39 ± 0.41	147.9 ± 47.5	108.8 ± 37.9
> 10 (g/dl) (*n* = 142)	2.03 ± 0.49	1.51 ± 0.46	165.6 ± 47.7	123.3 ± 40.3
Missing (*n* = 3, *n* = 4)				

*Nm*, Newton meter; *n*, number of patients; *BMI*, body mass index; *HCT-CI*, comorbidity index by Sorror; *KPS*, Karnofsky Performance Status; *allo-HCT*, allogeneic stem cell transplantation; *AML*, acute myeloid leukemia; *ALL*, acute lymphocytic leukemia; *MM*, multiple myeloma; *CML*, chronic myeloid leukemia; *MPS*, myeloproliferative syndrome; *MDS*, myelodysplastic syndrome; *MVIC*, maximal voluntary isometric contraction; *MIPT*, maximal isokinetic peak torque; *MET*, metabolic equivalent of task; *T_Therapie*, months between last therapy to allo-HCT; *T_Diagnosis*, months between last therapy to allo-HCT

## Discussion

Muscle function and performance in patients prior allo-HCT is considerably low. Our analysis in patients prior allo-HCT shows that two-third (66%) of the population have MVIC-values below the 50th percentile of the healthy reference group. Further, a considerable proportion of 84% for MIPT_Knee_ and 83% for MIPT_Hip_ are below the 50th percentile of the healthy reference group. Identified determinates for lower muscle strength were older age, lower BMI, being a women, and having a higher comorbidity-index (HCT-CI). Compared to the healthy reference values, sub-group analyses showed that patients with younger age and male gender possess highest deviations when comparing to other gender/age subgroups.

A few studies in the past have examined muscle strength in hematologic patients, including allo-HCT candidates demonstrated impaired muscle strength and function [8, 11]. Studies have shown significant respiratory and skeletal muscle weakness in patients after [7, 9] and prior allo-HCT treatment [8]. In comparison with reference values, significantly reduced muscle strength of the knee extensors assessed by handheld dynamometry were observed prior allo-HCT [26]. In addition to the muscle strength-based findings, it has been well described that cardiorespiratory fitness in allo-HCT candidates [4, 21, 27] is also significantly impaired prior transplantation showing the general physical deconditioning of allo-HCT candidates.

Compared to other cancer populations where stationary dynamometry has been applied, MVIC values in knee extensors, as a representative indicator muscle group for the locomotor function and for daily functional tasks [28], were higher than those of pancreatic cancer patients [29] (mean MVIC_Knee_ 159.7 Nm vs. 141.3 Nm respective 125.7 Nm). Similar results were reported for the MVIC for the hip flexors. With regard to the MIPT values, the results of our allo-HCT patients were also slightly higher [30]. When compared to breast cancer patients, our female subsample showed lower MVIC values of knee extensors, whereas the MIPT values are comparable or slightly higher depending on the treatment [17].

Presumably, there are multicausal reasons associated with reduced muscle strength in cancer patients and particular in allo-HCT transplant candidates. To explore the relevance of different potential reasons associated with muscle strength performance, we conducted a regression analysis for MIPT/MVIC of knee muscles. In general, the variance explaining characters of the different variables were comparable between models, showing same trends with small differences.

It is conceivable that various anti-cancer treatments (e.g., doxorubicin) and their adverse side effects negatively impact muscle mass and function [31]. Although we were not able to significantly detect this association within our regression analysis, some variables (time since last chemotherapy treatment and number of cardiotoxic treatment) showed borderline results, biologically plausible pathways have been reported, and findings from preclinical studies do exist [31]. Findings could be amplified by the fact that physical inactivity and inactivity increasing side effects are prevalent in cancer populations, further increasing the risk of degeneration of the locomotor muscles [32, 33]. As an example associations between impairments in contractile strength, muscle function, muscle power, and cancer-related fatigue with physical inactivity have been largely demonstrated [33]. The result is a vicious circle [34] in which it becomes increasingly difficult to perform activities of daily living.

With regard to gender differences, not surprisingly, our regression model showed that the values of the female gender are significantly lower. However, the deviation from the reference values indicates that men have considerably lower muscle strength than women. This finding is supported by observations that sarcopenia was more prevalent in men before allo-HCT [35].

A further possible reason for lower MVIC and MIPT could be related to hemodynamics respectively reduced hemoglobin levels. Kirkham and colleagues were able to show that muscle blood flow and O_2_ extraction adapt to compensate for chemotherapy-induced hemoglobin reduction during exercise with small muscle mass, but are not sufficient with large muscle mass [36]. In our analyses, hemoglobin was a marginally non-significant explaining factor of muscle strength for MIPT. Our descriptive report furthermore reveals a remarkable deviation in muscle strength, in the group with lower hemoglobin values (hemoglobin value MVIC − 11.9% and hemoglobin value MIPT − 13.3%) compared to those with higher hemoglobin values.

Our regression model did not show a significant influence of cardiotoxic treatment, as we could demonstrate for CRF [21]. Presumably, the reason for that is the nature of maximum strength testing procedure, only requiring short term local metabolic resources in comparison to CRF testing protocol [21, 27]. However, there is also evidence for a negative impact on the skeletal muscle due to cardiotoxic therapies [37] as well as glucocorticoid-induced myopathy [38, 39] and the general negative impact on muscle function of prior chemotherapies [40].

Significant influence was shown for higher age on lower muscle strength values. This is in line with observations, that muscle strength levels decrease with age. However, our results showed that the deviation from the healthy reference values was associated with patient age in that way and that younger patients were more affected by muscle strength loss than older patients. This is in line with the findings that an increasing proportion of older patients are eligible for allo-HCT and not only the age but possibly also the muscle strength status is crucial [10]. With increasing age, the higher prevalence comorbidities could also impact physical functioning of HCT patients [41]. This can be also seen in our regression models with the comorbidity index HCT-CI becoming a significant variance explaining character for MVIC_knee_.

It has been shown that sarcopenia and the loss of muscle mass as well as low muscle strength, with special emphasis on low knee extensor muscle strength values to predict overall survival and non-relapse mortality after transplantation [11], is associated with risk for treatment-related complications and poor prognosis in patients undergoing allo-HCT treatment [10, 11, 42, 43]. Furthermore, we are able to show that lower BMI values are predictive of lower muscle strength. This relationship is obvious on the one hand, but on the other hand, there is an association between low pretransplant BMI and poor clinical outcome after allo-HCT [44].

Based on the clinically relevant associations of muscle mass/function and prognosis after allo-HCT as well as our finding that muscle function seems to be generally impaired prior transplant, the revealed parameters in the regression analysis can be used for early identification and detection of patients with the goal to modify this risk parameter via interventions aiming to increase muscle mass.

First studies investigating so-called prehabilitative exercise interventions before allo-HCT already exist, demonstrating the feasibility of such approaches; however, larger RCTs are warranted to build robust knowledge on the effectivity of such approaches [45, 46].

A central strength of our study is the large sample of allo-HCT patients being investigated using gold standard muscle strength assessment procedures, limited by the factor of retrospective data collection obtained from available patient medical records. Furthermore, subgroup analyses of primary diagnosis were not adequately powered, and further studies are needed to provide specific estimation for high-risk patients of allo-HCT.

In conclusion, by applying gold standard assessment procedures, we were able to quantify in a rather large sample that muscle strength values are remarkably reduced directly prior allo-HCT. We can contribute to the existing knowledge that younger patients with male gender, lower hemoglobin-levels, and comorbidity burden are particularly affected by muscle impairments. Our data supports the approach of integration of early exercise programs (prehabilitation) to prepare for allo-HCT. However, more research is needed on further (clinical) factors playing a role in risk stratification prior allo-HCT and that may be influenced by physical exercise.

## Data Availability

No datasets were generated or analyzed during the current study.
